# Enhanced Blood Lymphocytes Apoptosis in Children with Inflammatory Bowel Disease

**DOI:** 10.1155/2013/415417

**Published:** 2013-08-29

**Authors:** M. A. El-Hodhod, R. H. Aly, S. R. Youssef, S. I. Mohamed

**Affiliations:** ^1^Department of Pediatrics, Faculty of Medicine, Ain Shams University, Cairo 11566, Egypt; ^2^Department of Clinical Pathology/Hematology, Faculty of Medicine, Ain Shams University, Cairo 11566, Egypt; ^3^Ministry of Health, Cairo 11566, Egypt

## Abstract

The aim of this work was to measure peripheral lymphocyte apoptosis during IBD flare and remission. *Subjects and Methods.* Flow-cytometric assessment of apoptosis of peripheral blood lymphocytes (PBL) was assessed in 30 children with IBD (16 with ulcerative colitis and 14 with Crohn's disease) compared to 22, age and sex matched, healthy children. This was carried out during a flare, whether in newly diagnosed or relapsing patients, and after achievement of remission. Clinical findings, complete blood count, liver transaminases, and kidney functions were assessed. *Results*. Early apoptotic and late apoptotic/necrotic lymphocytes were significantly higher during IBD flare compared to controls (*P* ≤ 0.01 and <0.01, resp., in ulcerative colitis and *P* ≤ 0.01 and <0.01, resp., in Crohn's disease patients). Remission values were significantly decreased but did not come back to the control levels. Early apoptotic values were significantly related to joint involvement in IBD patients (*P* < 0.0001). *Conclusions*. We can speculate a systemic nature of IBD as evident by enhanced peripheral lymphocyte apoptosis. This is related, to a great extent, to the disease process as it is more deranged in flare than in remission. Relation of this derangement to extraintestinal manifestations needs a special attention.

## 1. Introduction

Although the exact etiology of inflammatory bowel disease (IBD) still remains unclear, its pathophysiological mechanisms have been more extensively investigated. Nowadays, it is commonplace that in IBD, a wide diversity of immunological changes occurs, including altered populations of inflammatory cells and activation of a range of systemic inflammatory pathways [1–3]. Thus, Crohn's disease (CD) and ulcerative colitis (UC) should both be considered as systemic diseases being associated with clinical manifestations involving different organs outside the alimentary tract [4–6].

Apoptosis is a well-recognized process of cell death occurring as a series of changes in dying cells under several physiological conditions. On the other hand, necrosis occurs with cell death and empties its contents into the surroundings [7]. With starting apoptosis, phosphatidyl residues are externalized. Annexin V preferentially binds to phosphatidyl residues that can be identified by flow-cytometry [8].

Several studies have also addressed the role of apoptosis of intestinal lymphocytes in IBD. The noninflamed gut T cells have an increased susceptibility towards apoptosis that places a limit on the expansion of T cells and downregulates the mucosal immune response following direct or bystander stimulation by a specific antigen [9, 10]. In contrast, the inflamed gut T cells are resistant to apoptosis, exhibit a prolonged survival, and display an increased cytokine production that might in turn significantly aggravate the inflammation [11]. Increase in enterocyte apoptosis was proved by Di Sabatino et al. [12], while the increase in intestinal lymphocyte apoptosis was proved by Neurath et al. [13].

We hypothesize that peripheral blood lymphocyte apoptosis is enhanced in IBD patients as a consequence of the disease process. Hence, the aim of this work was to measure peripheral lymphocyte apoptosis during flare and remission among children with IBD. 

## 2. Methodology

This study was a cohort prospective study in which thirty children with IBD were included among patients who are followed up in the Pediatric Gastroenterology Clinic of the Children's Hospital, Ain Shams University, Cairo, Egypt. Diagnosis of IBD was based on the Porto criteria [14]. They were 22 males and 8 females with an age range of 3.5–16 years (mean = 11.1 ± 3.5 years). They included 16 patients with UC and 14 patients with CD. Twenty-two, age and sex matched, healthy children were recruited among healthy relatives to serve as a control group. They were 13 males and 9 females, with an age range of 3–16 (mean = 11.8 ± 3.5 years).

An informed consent was taken from each guardian after obtaining the approval of the ethics committee of the Children's Hospital, Ain Shams University. 

### 2.1. Inclusion Criteria

1. IBD patients with an age range between 2 and 18 years.
2. Enrollment during a disease flare whether as a first presentation or as a relapse. Disease flare was assessed using Pediatric Ulcerative Colitis Activity Index (PUCAI) for UC [15] and modified pediatric Crohn's disease activity index (PCDAI) for CD [16].

### 2.2. Exclusion Criteria

1. Patients with evidence of systemic infections to avoid infections' impact on apoptosis.
2. Patients who are currently (or in the last 2 months) taking steroids or any immune modulating drugs.
3. Patients suffering from other concomitant immunological nonrelated disorders.

All included patients were assessed in activity, and they were reassessed after achieving disease remission. They were subjected to a detailed medical history with a focus on gastrointestinal symptoms. Presence of extraintestinal manifestations during flare were asked for including glossitis, conjunctivitis, scleritis and episcleritis, pulmonary involvement, erythema nodosum, renal stones, jaundice, joint related symptoms, and weight loss. Careful clinical assessment with special emphasis on abdominal and rectal examination was done. Arthritis was diagnosed clinically with pain, swelling, and hotness of joints. Mere pain, limited to joint space, was diagnosed as arthralgia whether continuously or intermittently reported. Laboratory workup included the determination of liver and kidney functions (Synchron CX-5 delta of Beckman Instruments Inc., Scientific Instruments Division, Fullerton, CA, USA). Complete blood count (Coulter T660, Miami, USA) and flow-cytometric assessment of apoptotic peripheral lymphocytes were done using a TACS Annexin V-fluorescein isothiocyanate (FITC) detection kit (TACS, Trevigen Inc., USA). For the detection of apoptosis, mononuclear cells were harvested, washed, and then dually incubated for 15 minutes with Annexin V-FITC and propidium iodide. This combination allows the differentiation between early apoptotic cells (EAC) which are Annexin V positive and late apoptotic (LA) and/or necrotic cells (NC) which are both Annexin V positive and propidium iodide positive.

The absolute counts of lymphocytes and their percent were quantified using white blood cells obtained from the complete blood picture and flow-cytometric analysis.

The percent of apoptotic and viable lymphocytes was obtained from their corresponding flow-cytometric histograms. Then, their absolute values were calculated against the absolute lymphocytic count using the following equations.

1. Absolute lymphocytic count = (percent of lymphocytes × total WBCs)/100.
2. Absolute apoptotic lymphocytic count = (percent of apoptotic lymphocytes × absolute lymphocytic count)/100.
3. Absolute viable lymphocytic count = (percent of viable lymphocytes × absolute lymphocytic count)/100.

### 2.3. Data Management and Analysis

The collected data were analyzed using Statistical Package for Social Science (SPSS 15.0.1 for windows; SPSS Inc., Chicago, IL, 2001). Student's *t*-test was used to compare mean values of dependent and independent variables. Linear regression analysis was used to determine magnitude of impact of different parameters on the occurrence of joint involvement. 

## 3. Results

### 3.1. Clinical Characteristics at Enrollment

The patients' mean age was 11.1 ± 4 years on presentation with a male predominance (10/16 of UC patients and 12/14 of Crohn's disease patients). The weight (expressed as a percent from the 50th centile for age) was 77.5 ± 13.8% in the UC patients and 57.3 ± 11.5% in CD patients. Bleeding per rectum was a presenting symptom in 9/16 of UC patients and 8/14 of CD patients. Abdominal pain was documented in 15/16 of UC patients and 12/14 of CD ones. Diarrhea was recorded in 15/16 of UC patients and 10/14 of CD ones. Fever was recorded in 8/16 of UC patients, while it was present in 10/14 of the CD patients. Arthritis was seen in 3 patients within the UC group (18.75%) and in 2 patients in the CD group (14.29%). Arthralgia was reported in 4 patients in UC group (25%) and 5 patients in the CD group (35.71%). No other extraintestinal manifestations were noticed in this series during the period of the current study. No organomegaly was detected in our patients groups. Rectal examination did not show any perianal inflammation, granulomas or skin tags, anal fissures, and rectal polyps or masses.

### 3.2. Laboratory Parameters of Studied Groups (Table 1)

In the present study, hemoglobin concentration was significantly lower (*P* ≤ 0.05) in UC and CD patients during flare compared to controls (*t* = −5.19 and −5.88, resp.). It was also significantly increased (*P* ≤ 0.05) during remission but not the control levels (*t* = −3.26 and −4.42, resp.). TLC did not show any significant differences (*P* ≥ 0.05) between the patients groups and controls neither in flare nor in remission. Significantly higher (*P* ≤ 0.05) mean values of platelets count were recorded in CD patients in both flare (*t* = 2.88) and remission (*t* = 4.06) when compared to controls. However, platelets count did not show any significant differences (*P* ≥ 0.05) in UC patients and controls in both flare and remission. Erythrocytes sedimentation rate was significantly higher (*P* ≤ 0.05) in UC and CD patients during flare compared to controls (*t* = 12.18 and 23.85, resp.). It was significantly lower (*P* ≤ 0.05) during remission but not to the control levels (*t* = 9.61 and 8.88, resp.).

Serum ALT levels were significantly higher (*P* ≤ 0.05) in patients with UC and Crohn's disease compared to controls in both the flare (*t* = 4.37 and 2.30, resp.) and the remission (*t* = 5.11 and 3.92, resp.), yet mean values of these enzymes were within normal ranges.

Similarly, AST serum levels were significantly higher (*P* ≤ 0.05) in patients with UC and Crohn's disease compared to controls in both the flare (*t* = 7.95 and 4.48, resp.) and the remission (*t* = 7.42 and 5.05, resp.), yet mean values of the enzyme were within normal ranges.

Serum creatinine mean levels showed no significant differences (*P* > 0.05) in both patients groups in both flare and remission phases compared to the control group.

The current study showed that in both UC and CD patients, EAC% and LAC/NC% were significantly higher compared to control subjects. All the apoptotic indices measured including EAC% and LAC/NC% showed no significant differences among UC patients compared to CD patients (Table 2). 

Within patients of UC, EAC% were significantly higher in the flare phase compared to the remission phase, whereas LAC/NC% did not show any significant differences between UC patients in the flare compared to the remission phases (Table 3).

Within patients of CD, EAC% and LAC/NC% were both significantly higher in the flare compared to the remission phase (Table 3). 

**Figure 1:**
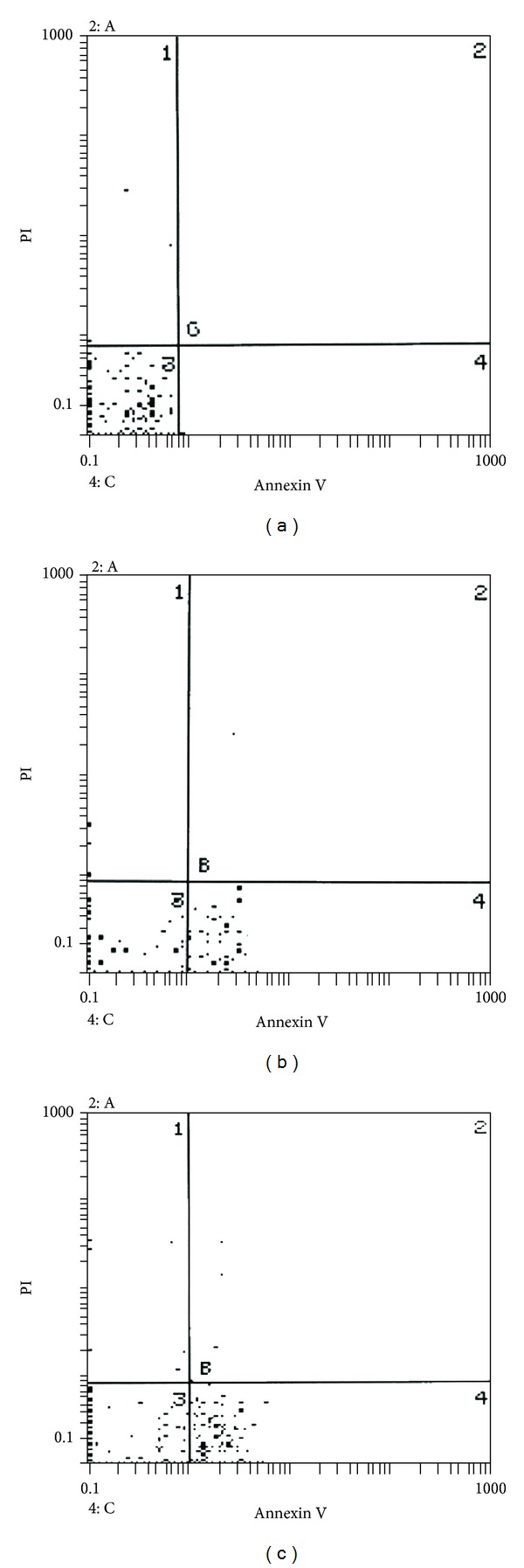
(a) Showing apoptosis in peripheral blood lymphocytes by flow-cytometry using Annexin V and propidium of one of the controls. The viable cells are 99%. The early apoptotic cells are 0.29%, the late apoptotic cells are 0.06%. (b) Showing apoptosis in peripheral blood lymphocytes by flow-cytometry using Annexin V and propidium of an ulcerative colitis patient at flare. The viable cells are 52.2%. The early apoptotic cells are 47.3%. The late apoptotic cells are 0.2%. (c) Showing apoptosis in peripheral blood lymphocytes by flow-cytometry using Annexin V and propidium of a Crohn's disease patient at flare. The viable cells are 51.4%. The early apoptotic cells are 47.7%. The late apoptotic cells are 0.58%.

The distribution of peripheral blood lymphocytes is represented in 4 quadrants of the dot plot, which is set so that the *x*-axis reflects the Annexin V-FITC florescence, and the *y*-axis reflects the propidium iodide florescence. Density scale is used to determine the number of cells in each quadrant. A population of cells that are negative for both Annexin V and propidium iodide is in the lower left quadrant of dot plot. These are normal viable cells. The lower right quadrant cells take Annexin V-FITC (EAC%); upper right and left quadrants cells take both dyes (LA% and/or NC%) (Figures 1(a), 1(b), and 1(c)).

Joint affection was the only example of systemic involvement in our series of IBD during this study. It was tested in a linear regression analysis model against multiple variables including age, type of IBD (UC or CD both during flare), ESR, hemoglobin concentration, platelet count, early apoptotic lymphocytes, and late apoptotic/necrotic lymphocytes. At *R* = 0.862,  *R* ^2^ = 0.743, adjusted *R* ^2^ = 0.661, and *P* < 0.0001, early apoptotic index (Beta = 0.64, *P* < 0.0001), ESR (Beta = −0.34 and *P* = 0.02), and type of IBD (Beta = 0.354 and *P* = 0.023) were the only significant determinants of this involvement.

## 4. Discussion

The results of the current study revealed significantly enhanced apoptosis of circulating lymphocytes among IBD patients during flare compared to remission. Both values were significantly higher than controls. These results could be interpreted in view of other studies which reported systemic involvement in the pathogenesis of IBD [17, 18]. This may reflect the possibility of being a systemic disease.

Danese et al. [19] and Fava and Danese [20] discussed the pathogenesis of extraintestinal manifestations in IBD patients and reported the crucial role of enteric flora in activating the immune system against bacterial antigens and contemporary against colonic mucosa on the basis of an antigenic cross-reactivity (antigen mimicry). They hypothesized that the sharing of these colonic antigens by extraintestinal organs, associated with a genetic susceptibility, would finally lead to an immune attack to these organs. One of the best examples offered was represented by primary sclerosing cholangitis occurring in UC: in a subset of patients where the presence (in sera and colonic mucosa) of anticolonic mucosa autoantibodies that cross-react with biliary epithelium has been identified [21]. Furthermore, a colonic epithelial protein and the human tropomyosin isoform, which are not only expressed in the colon but also in the biliary tract, skin, eyes, and joints [22], have been suspected to be the major common targets of autoimmune attack in extraintestinal organs of IBD patients.

Normal homeostasis of the immune system is controlled by a balance of production and death. During an immune response, homeostasis is disturbed as antigen-presenting cells become activated and promote the clonal expansion of antigen-specific lymphocytes. Shortly after the peak of the response, controlled induction of apoptosis, of both antigen-presenting cells and lymphocytes, restores homeostasis. This process is critical to ensure protective immunity and avoid lymphoid neoplasia and autoimmunity [23].

Apoptosis plays a critical role in lymphocyte development and homeostasis. Enhanced lymphocyte apoptosis can cause immunodeficiency through cell loss. Conversely, inhibition of apoptosis can lead to the development of autoimmunity or lymphoma. Two major pathways contribute to the regulation of lymphocyte cell death, death by neglect and death by instruction [24].

Apoptosis plays an important role in downregulation of the inflammatory response, for example, by reducing the lifespan of activated lymphocytes [25]. Increased circulating lymphocytes apoptosis could also be considered as a protective mechanism against organ injury as increased lymphocyte apoptosis was found to be linked to anti-inflammatory cytokines secretion and thereby may contribute to preventing unwanted immune response and organ injury [26, 27]. O'shea et al. [28] reported that TNF influenced the function of antigen-presenting cells (APCs), but, again, its effects are complicated. In some circumstances, TNF can activate APCs, augment antigen presentation capability, and upregulate the expression of costimulatory molecules. However, it can also inhibit the function of mature lymphocytes and might induce their apoptosis and impair antigen presentation, to prevent the organ damage. Researchers have shown that apoptotic cells actively suppress the inflammatory response [29, 30]. Fadok et al. [29] reported that uptake of apoptotic versus necrotic cells caused the macrophage to respond in an anti-inflammatory or proinflammatory manner, respectively. Voll et al. [30] demonstrated that addition of apoptotic lymphocytes to endotoxin-stimulated PBMC caused a shift from secretion of proinflammatory cytokines (TNF-*α*, IL-1*β*, and IL-12) to anti-inflammatory cytokines (IL-10). 

Increased circulating lymphocytes apoptosis might also be attributed to nutrients deficiency which is very common in IBD patients [31–36].

The triggering event for the activation of the immune response in IBD has yet to be identified. Possible factors related to this event include a pathogenic and nonpathogenic organism [37], an immune response to an intraluminal antigen (e.g., protein from cow milk) [38], or an autoimmune process whereby an appropriate immune response to an intraluminal antigen [39]. 

The overall frequency of arthritis in our series was 5/30 (16.67%). Gravallese and Kantrowitz reported that frequency of peripheral arthritis in IBD ranges from 17 to 20%, and it is more common in CD [40]. In a retrospective study that included 1459 patients with IBD, peripheral arthritis was present in 6% of patients with UC and in 10% 0f patients with CD [41]. Another study showed arthritis in 10% in a group of patients with IBD [42]. Arthritis development in the current study was found to be predominantly related to the increase of early lymphocyte apoptosis especially in the ulcerative colitis group. This reflects that deranged lymphocyte apoptosis may be a significant player in the pathology of IBD with special reference to development of extraintestinal manifestations.

Our results might be augmented with the findings of Veltkamp et al. [43], who suggested that increased apoptosis of Treg cells plays a potentially important role in the pathogenesis of IBD and can be reversed by anti-TNF*α* treatment. 

In conclusion, we can speculate a systemic nature of IBD as evident by enhanced peripheral lymphocyte apoptosis. This is related to a great extent to the disease process as it is more manifested during flare than in remission. Failure to normalize in remission may reflect incomplete control or involvement of other working mechanisms. Relation of this derangement to extraintestinal manifestations needs special attention.

## Figures and Tables

**Table 1:** Laboratory parameters in studied groups.

	UC flare mean (±SD)	UC remission mean (±SD)	CD flare mean (±SD)	CD remission mean (±SD)	Control mean (±SD)
TLC (10^3^/L^−6^)	6.7 ± 1.4	6.5 ± 1.5	8.9 ± 1.8*	8.2 ± 0.6	7.7 ± 2.3
Lymphocytes%	39.2 ± 11.5	35.9 ± 12.9	38.5 ± 19.1	39.5 ± 16	45.8 ± 15.3
Hemoglobin (gm/dL)	9.6 ± 2.2*	11.2 ± 1.2*	9.8 ± 1.5*	10.5 ± 1.6*	12.3 ± 0.7
Platelets (10^3^/L^−6^)	249 ± 63	257.3 ± 107.6	352.6 ± 175.4*	346.5 ± 61.1*	280 ± 31
ESR (mm/hr)	33.4 ± 8.9*	30.2 ± 11.2*	41.1 ± 5.7*	34.3 ± 12.3*	7.6 ± 3.1
ALT (u/L)	26.8 ± 4.4*	27.9 ± 4.0*	22.5 ± 7.1*	26.5 ± 2.2*	17.9 ± 6.2
AST (u/L)	33.9 ± 3.8*	33.0 ± 3.6*	28.7 ± 8.0*	30.1 ± 5.3*	17.9 ± 6.9
Creatinine (mg/dL)	0.39 ± 0.1	0.3 ± 0.1	0.27 ± 0.04	0.23 ± 0.04	0. 32 ± 0.14

Test of significance used is *t*-test for independent samples. *P < 0.05: significant in the patients groups in relation to controls.

UC: ulcerative colitis; CD: Crohn's disease.

**Table 2:** Comparison of apoptotic indices in ulcerative colitis and Crohn's disease patients (in both flare and remission phases) and the controls.

	UC (I) mean (±SD)	CD (II) mean (±SD)	Control (III) mean (±SD)	I versus II *t* (*P*)	I versus III *t* (*P*)	II versus III *t* (*P*)
EAC flare	33.89 ± 8.49	27.71 ± 14.75	2.82 ± 7.02	1.33 (0.013)	11.92 (<0.001)	6.69 (<0.001)
EAC remission	20.83 ± 11.88	17.41 ± 13.59	2.82 ± 7.02	0.64 (0.52)	5.46 (<0.001)	4.03 (<0.001)
LAC and/or NC flare	3.29 ± 2.82	4.09 ± 2.76	0.30 ± 0.45	−0.73 (0.49)	4.89 (<0.001)	6.26 (<0.001)
LAC and/or NC Remission	2.07 ± 2.90	1.09 ± 0.95	0.30 ± 0.45	1.02 (0.31)	2.83 (<0.001)	3.19 (<0.001)

EAC: early apoptotic cells; LAC/NC: late apoptotic/necrotic cells.

**Table 3:** Comparison of apoptotic indices in patients in flare compared to remission.

	Flare mean (±SD)	Remission mean (±SD)	*t*-test	*P *
UC				
EAC%	33.89 ± 8.49	20.83 ± 11.88	5.3779	<0.0001
LAC and/or NC%	3.29 ± 2.82	2.07 ± 2.90	1.043	0.317
CD				
EAC%	27.71 ± 14.75	17.41 ± 13.59	2.393	0.04
LAC and/or NC%	4.09 ± 2.76	1.09 ± 0.95	3.246	0.01

EAC: early apoptotic cells; LAC/NC: late apoptotic/necrotic cells.
